# Synthesis, characterization, and the influence of energy of irradiation on optical properties of ZnO nanostructures

**DOI:** 10.1038/s41598-022-24648-x

**Published:** 2022-11-21

**Authors:** Ghazouan Mahmood Abdelghani, Ali Ben Ahmed, Aseel Basim Al-Zubaidi

**Affiliations:** 1 Laboratory of Applied Physic, Department of Physics, Faculty of Science of Sfax, University of Sfax, Sfax, Tunisia; 2 Department of Materials Engineering, University of Technology, Baghdad, Iraq

**Keywords:** Chemistry, Materials science, Nanoscience and technology, Optics and photonics, Physics

## Abstract

Zinc oxide nanoparticles (NPs) are synthesized by the sol–gel method for optoelectronics, photonic, and medical applications. The as-synthesized NPs are irradiated with lasers with wavelengths of 1064 nm and 532 nm in an attempt to improve the particles' structural and functional properties. The NPs are characterized by means of X-ray diffraction, scanning and transmission electron microscopy techniques, atomic force microscopy, and ultraviolet–visible spectroscopy. The structural analysis shows that the particles have a polycrystalline hexagonal structure of the space group (P6_3_mc). The average crystallite size of nanoparticles varies in the range of 13–15 nm after and before irradiation by a laser beam. The morphological characterization revealed the formation of agglomerates of NPs in the range of 42–46 nm A shift is observed in the absorption spectra after irradiation with a laser beam. This shift is due to the effect of irradiation on the sample’s electronic structure. This effect is confirmed by a decrease in the bandgap energy. The optical parameters are also deduced and discussed. The irradiation of ZnO nanoparticles by laser beams of wavelength 1064 nm and 532 nm decreases the size of the crystallites which increases their antibacterial activity. The biological activity of the NPS and in improving the efficacy of antibiotics are tested and analyzed. Results emphasized the positive role of ZnO-NPs in the above-mentioned application.

## Introduction

Nanoparticles and nano-powders have attracted the attention of scientists, engineers and medical doctors due to their versatile applications and promising advantages for future applications and requirements^1–7^. ZnO-NPs can strongly ability to trigger oxygen species production and release zinc ions. This, in turn, may induce cell apoptosis. In addition, ZnO-NPs are known to maintain the integrity of insulin. Due to the strong absorption property in the UV region, ZnO-NPs have been used as an anti-aging element and in cosmetics. Nevertheless, ZnO-NPs are receiving increasing attention from the scientific community for being used as anticancer and antibacterial agents. As compared with other nanoparticles, ZnO-NPs are less expensive and less toxic when employed for biomedical applications^8^. The antibacterial agents may be classified into organic and inorganic agents. Accordingly, ZnO, as an inorganic material, has proved to be a superior antibacterial agent. Nevertheless, the antibacterial activity of ZnO NPs against Gram-positive and Gram-negative bacteria has already been identified^9^. In addition, ZnO NPs were identified to have an antibacterial effect against spores^10^. It is well known nowadays that the ZnO activity depends greatly on its particle size, thus its surface area, and concentration. On the other hand, the quality of its crystal structure and shape has little effect^11^. Umair Manzoor et al. investigated, the effect of the synthesis parameters (namely, temperature, nucleation time, and post-synthesis heat treatment) on the sensing properties of ZnO NPs^12^. These parameters were found to affect the particle sizes and their crystallinity tremendously. The ZnO NPs were synthesized by a co-precipitation method using zinc acetate, potassium hydroxide, and methanol as a solvent medium. However, the particles sizes of the ZnO powders were ranging between $100 nm$ and $458 nm$ depending on the synthesis temperature. Several works in the literature reported the successful preservation of the inherent selectivity of the ZnO-NPs to cancerous cells by synthesis of these materials using precipitation method^13,14^. Sol–Gel method is a novel method for the synthesis of ZnO-NPs^15,16^. Yin Zhang et al. reported, in 2013, on the biomedical applications of ZnO nanomaterials such as biomedical imaging, drug and gene delivery, and bio-sensing^17–19^.

In this paper for the first time, we present all experimental results on the synthesis of ZnO nanoparticles via the sol–gel method, and the characterization of the nanostructure, morphologies analysis, and optical properties before and after irradiation by 1064 nm, and 532 nm. The effect of sonication on effective mean diameter of prepared ZnO nanoparticles are analyzed and discussed. The effect of laser irradiation on the crystallite size, absorption spectra, optical bandgap, refractive index, extinction coefficient, absorption coefficient, and optical conductivity of ZnO nanoparticles, will be reported and discussed. The antibacterial activities of ZnO nanoparticles will also be reported and discussed.

To our knowledge, no papers have reported and discussed the effect of laser irradiation on nanostructure properties, morphologies analysis, and optical properties of the Zinc oxide nanoparticles.

## Experimental details

### Chemical synthesis of ZnO NPs

The ZnO nanoparticles were synthesized by sol–gel method. In this method zinc acetate dihydrate (Zn(CH_3_CO_2_)_2_.H_2_O) and sodium hydroxide (NaOH) were used as precursors without further purification. Stoichiometric amount of zinc acetate was added to sodium hydroxide solution at molar ratio of 1:85. The solution was stirred continually for 4 h while the temperature was maintained in the range 70–75 °C. The product is then filtered using Whatman filtering papers. The precipitate was then washed with ethanol and deionized water several times to remove the undesired impurities (i.e. the sodium salt ${\mathrm{NaCH}}_{3}{\mathrm{CO}}_{2}$) and to ensure high degree of purification. This was then followed by drying at 90 °C for 2 h. The dry precipitate was subjected to ball milling to produce fine ZnO nanoparticles.

### Characterization of synthesized ZnO NPs

The X-ray diffraction patterns were measured for the ZnO nanoparticles before and after being radiated with Nd-YAG lasers. All the patterns were measured in the range of 5–80 (2θ). The Cu Kα-radiation having wavelength of 1.54060 Å. The surface morphology of synthesis ZnO NPs before and after irradiation were analyzed by the scanning electron microscopy. Further investigation of the nanoparticles morphology and inner structure was accomplished by means of transmission electron microcopy. The topographic features of the synthesized nanoparticles were examined by using the atomic force microscope. The FTIR spectroscopy measurements were accomplished using TENSOR27 an FTIR spectrometer from Bruker. All the absorption spectra measurements were recorded using a continuous IR light in the wavenumber range of 4000–600 cm^−1^. The optical absorption spectra of ZnO NPS before and after irradiation were obtained by UV–Vis spectroscopy in the range 190–1100 nm. The efficacy/biological activity of the ZnO nanoparticles before and after irradiation by laser beam were tested.

## Results and discussions

### Effect of energy irradiation on the structure analysis

**Figure 1:**
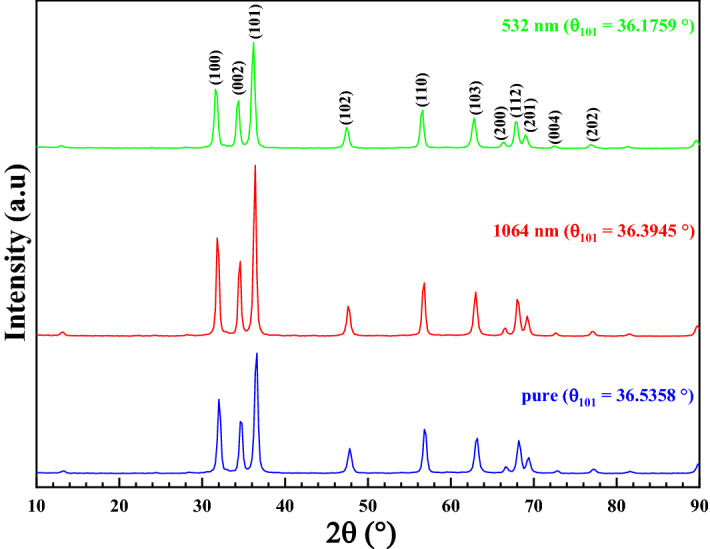
The XRD patterns before and after irradiation with laser beams for ZnO NPs.

The Fig.1 shows the XRD patterns for the ZnO nanoparticles before and after being irradiated with laser beams having wavelengths of 532 nm and 1064 nm. The laser beam used in the present work is the Neodymium-Yttrium Aluminum Garnet (Nd-YAG) laser. All the diffraction peaks identified in the patterns could be indexed to a single phase of the hexagonal crystal structure of space group P6_3_mc and space group number 186 (ICDD reference number (98-015-5780)^20^. The most preferred orientation of the crystal structure is the $(101)$ plane as shown from the highest peak intensity. The ZnO nanoparticles shows no change in the preferred orientation after irradiation with laser, while a small shift of the peak position towards low diffraction angles is observed when increasing the laser irradiation energy. No additional peaks are observed after irradiation with laser, indicating the high crystalline quality of the material.

There are two lattice parameters for hexagonal unit cell, known as ‘*a*’ and ‘*c*’ which can be calculated from XRD spectra of samples by the following equation^21^:

1 $$ \frac{1}{d} = \frac{4}{3}\left( {\frac{{h^{2} + k^{2} + hk}}{a}} \right) + \frac{l^2}{{c^2}} $$

where $$h, k $$ and $$l$$ are known as the Miller index. $$d$$ interplanar distance of the crystal.

The average crystallite size $D$ of the nanoparticle of ZnO was estimated by using the standard equation known as the Scherrer formula^22^:

2 $$ D = \frac{0.9\lambda }{{\beta \cos \left( \theta \right)}} $$

where $\lambda = 1.5406{\text{ {\AA}}}$ is the Cu kα line, $\theta$ is the Braggs’ XRD diffraction angle and $\beta$ is the full width at half maximum (FWHM) in radian.

The average crystallite size was exhibited in Table 1. The average crystallite size of nanoparticles (D) is equal to 13.4198 nm before irradiation, while this value increases to 14.3710 nm after irradiation with 1064 nm laser beam and decreases to 13.4708 nm after irradiation with 532 nm laser beam. The slight variation in crystallite size after irradiation with the 532 nm and 1064 nm lasers leads to slight expansion and deformation of the samples due to thermal effect caused by the energy of laser beam^23^.

**Table 1:** ZnO crystallite size before and after irradiated with lasers having wavelengths of $532 nm$ and $1064 nm$.

Samples	hkl (1 0 1)					Grain size D_TEM_ (nm)
	2θ (°)	β (deg.)	D_XRD_ (nm)	$\delta (\times {10}^{-4} {nm}^{-2})$	$\varepsilon (\times {10}^{-4} )$	
pure	36.5358	0.62340	13.4198	55.5274	25.8300	46
Irradiated by 1064 nm	36.3945	0.58190	14.3710	48.4201	24.1203	45
Irradiated by 532 nm	36.1759	0.62040	13.4708	55.1078	25.7322	42

The dislocation density for the prepared samples has been studied by the following relation^24^:

3 $$  \delta = \frac{1}{D^2} $$

The lattice strain for each prepared samples was calculated by the equation:

4 $$ \varepsilon = \frac{\beta \cos \left( \theta \right)}{4} $$

The complete structural analysis for pure and irradiated ZnO NPs has been listed in Table 1, the variation in the crystallite size, dislocation density and lattice strain were observed with respect to pure ZnO. These clearly indicate the effect of the laser irradiation energy on the structural properties of the prepared ZnO NPs.

The lattice parameters (a and c) for ZnO nanoparticles were calculated using the following Eq. (5) and (6) were revealed in Table 2:

5 $$ a = \frac{\lambda }{{\sqrt 3 \sin \theta_{100} }} $$

6 $$ c = \frac{\lambda }{{\sin \theta_{002} }} $$

where $\theta_{100}$ and $\theta_{002}$ are the diffraction peaks angles of the $\left( {100} \right)$ and $\left( {002} \right)$ planes respectively.

**Table 2:** ZnO samples Lattice parameters (c, a) before and after irradiated with lasers having wavelengths of $532 nm$ and $1064 nm$.

Samples	2θ_100_ (°)	2θ_002_ (°)	a (Å)	c (Å)	c/a	V(Å^3^)
pure	32.0606	34.7003	3.221	5.166	1.6038	46.4145
Irradiated by 1064 nm	31.8947	34.5445	3.237	5.188	1.6027	47.0764
Irradiated by 532 nm	31.6961	34.3471	3.257	5.217	1.6017	47.9263

As can be seen Table 2, the analysis of the XRD pattern indicates that the constants of the crystal structure are such that *a* = 3.221Å, *c* = 5.166 Å for pure ZnO. After irradiation by $1064 nm$ laser beam, these parameters show a small increase (a = 3.237 Å, c = 5.188 Å). While, after irradiation by 532 nm laser beam, these parameters show a remarkable increase (a = 3.257 Å, c = 5.217 Å). This is due to the shift in the diffraction angle under the effect of irradiation.

The unit cell volumes could also be calculated through Lattice Geometry as given below^25^:

7 $$  V = \frac{\sqrt 3 a^2c}{2} = 0.866a^{2} c $$

The length of the Zn–O bond was calculated using equation:

8 $$  L = \sqrt {\left[ {\frac{a}{3} + c^2\left( {0.5 - \mu } \right)^{2} } \right]} $$

where $\left( \mu \right)$ is the positional parameter of the wurtzite structure that indicates the extent of atoms displacement relative to the following plane in the c axis, as expressed with equation:

9 $$  \mu = \frac{a^2}{{3c^2}} + 0.25 $$

It is indeed interesting to note that the variation of $V$ (Table 2) with the energy of irradiation shows the trend that is similar to the variation of $L$ (Table 3) with the energy of irradiation.

**Table 3:** Zn–O bond lengths, and the inter-planer d-spacing of ZnO nanoparticles before and after irradiated with lasers having wavelengths of $532 nm$ and $1064 nm$.

Samples	hkl (1 0 1)	d_Bragg_ (Å)	d_theo_ (Å)	μ	L (Å)
	**2θ (°)**				
Pure	36.5358	2.4574	2.7332	0.3795	1.9610
Irradiated by 1064 nm	36.3945	2.4666	2.7462	0.3797	1.9703
Irradiated by 532 nm	36.1759	2.4810	2.7628	0.3799	1.9820

The plane d-spacing $\left( d \right)$ associated with the lattice parameters $\left( {a, c} \right)$ and the Miller indices $\left( {h k l} \right)$ concisely predictable in Table 3 is calculated via the theoretical and Braggs’ equations as well^26–28^:

10 $$  d_{theo} = \frac{1}{{\sqrt {\left[ {\frac{{h^{2} + hk + k^2}}{a}} \right] + \frac{l^2}{{c^2}}} }} $$

11 $$ d_{Bragg} = \frac{\lambda }{2\sin \theta } $$

The d-spacing values obtained from the Braggs’ law, and the theoretical equations, are almost identical. We also note the increase in the d-spacing with the increase in the irradiation energy. This is caused by the variation in volume and Zn–O bonds length by increasing of energy of laser beam.

### Effect of energy irradiation on the morphology of ZnO NPs

#### Scanning electron microscopic analysis

**Figure 2:**
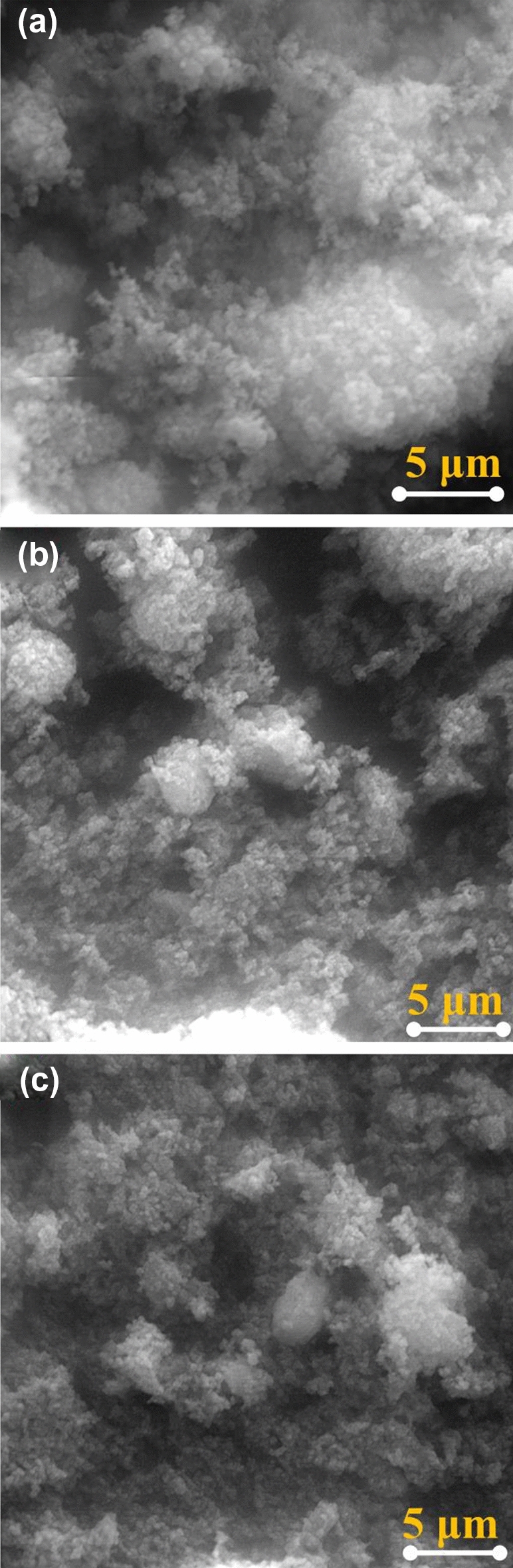
The SEM images of the ZnO powders before (**a**), and after irradiation with laser having wavelength of 1064 nm (**b**), and 532 (**c**).

The morphology of the nanoparticles was examined under the scanning electron microscope at the highest available magnification. Figure 2 show the scanning electron micrographs of the ZnO nanoparticles before (Fig. 2a), after the irradiation with a laser having wavelengths of $1064 nm$ (Fig. 2b), and after the irradiation with a laser having wavelengths of $532 nm$ (Fig. 2c). The irradiation duration was 1 min, The pulse duration is equal one nano second for three particles with Nd: Yag laser pulses. The three nanoparticles investigated show the formation of nanoparticles of well-established uniformity of size and morphology. The formation of nanoparticles is also characterized by a high tendency of these particles to agglomerate. This property is a characteristic of tiny particles which is attributed to the van der Waals forces at low dimensions. Furthermore, the nature of the particle’s topography decides the degree of these forces. The presence of asperities is known to increase the van der Waals forces due to an increase in mechanical interlocking between the closely adjacent particles. XRD relative intensity of each diffraction face on ZnO pure (9176) and after irradiated by a laser having wavelengths of $532 nm$ (8092) and $1064 nm$ (13,024) changes according to crystal morphology.

#### Transmission electron microscopic analysis

**Figure 3:**
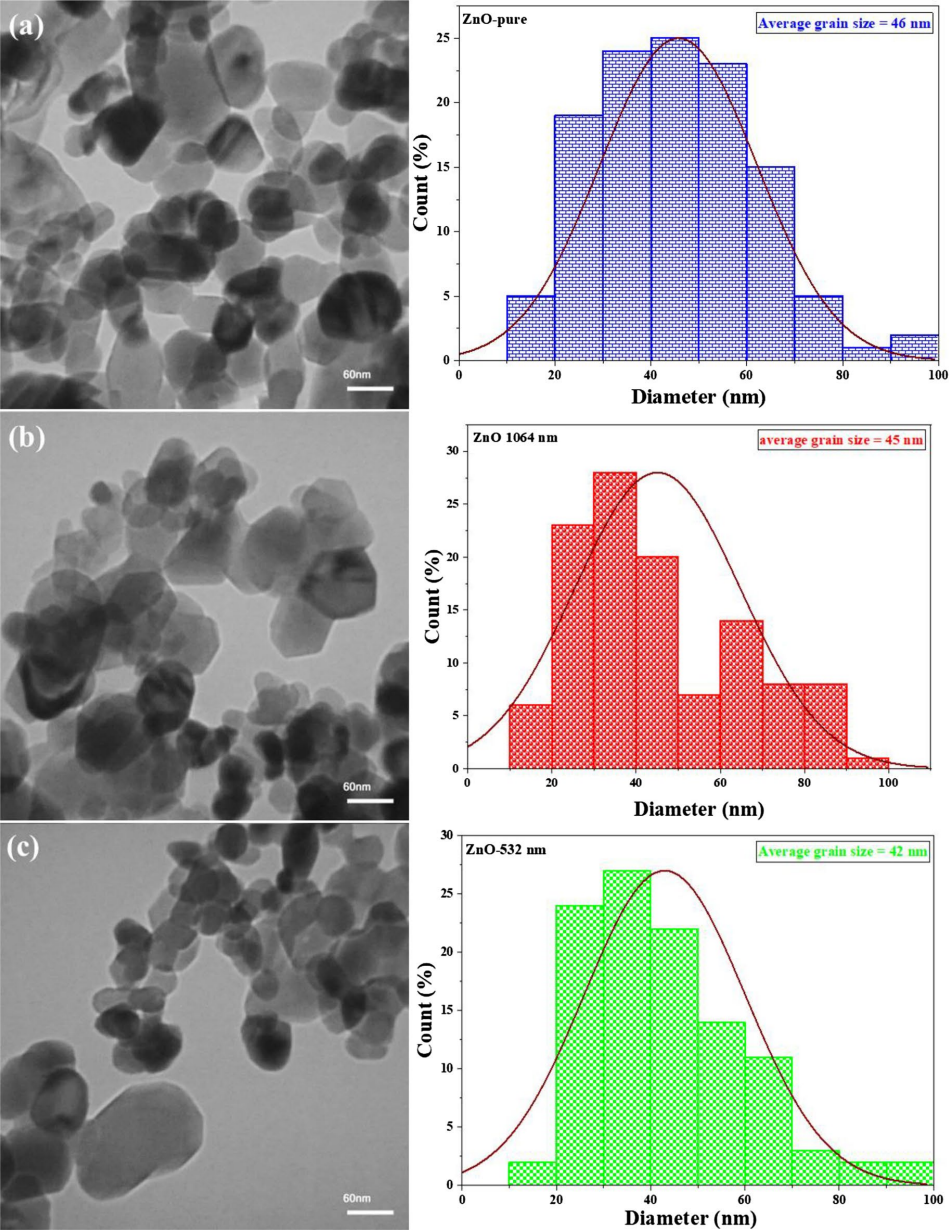
The TEM images and corresponding particle size histogram of the ZnO powders before (**a**) and after irradiation with laser having wavelength of 1064 nm (**b**), and 532 nm (**c**).

In order to confirm the morphological as well as the structural properties of the nanoparticles, transmission electron microscopy was used. The TEM image of the ZnO nanoparticles, and the corresponding particle size histogram are shows in Fig. 3a before the irradiation with a laser, in Fig. 3b after the irradiation with a laser having wavelengths of $1064 nm$, and in Fig. 3c after the irradiation with a laser having wavelengths of $532 nm$. The TEM images of the ZnO nanoparticles exhibits nanoparticles having an octahedral shape corresponding to the spinel lattice structure which was identified from the analysis of the XRD patterns. As can be seen Table 1, the sizes identified are in the range of $42-46 nm$.

### Effect of energy irradiation on the particle size distributions

All the nanoparticles were initially analyzed after adding a small amount of the nanoparticles to the dispersion solution. Sonication is a process in which sound waves are used to agitate particles in a medium. Sonicators either produce sound waves in a water bath, where nano particles (10–30) gm are placed in beaker for (10–15) minute. Sonication is performed to achieve multiple purposes such us deagglomeration of nanoparticles, size reduction, and dispersion in the base fluid. Then the measurements were repeated after the sonication of the specimens for an extended period of time. The measurement parameters are shown in Table 4, where water was used as the dispersion solution and a laser beam of $660 nm$ wavelength was used in the measurement.

**Table 4:** The parameters of the particle size distribution measurements.

Measurement parameters			
Temperature (°C)	25.0	Runs completed	3
Liquid	Water	Run duration	00:00:30
Viscosity (cP)	0.890	Total elapsed time	00:01:30
Reference index Fluid	1.330	Average count rate (kcps)	471.7
Angle (°)	90.00	Reference index real	1.590
Wavelength (nm)	660.0	Reference index imaginary	0.000
Baseline	Auto (slope analysis)	Dust filter	Off

**Figure 4:**
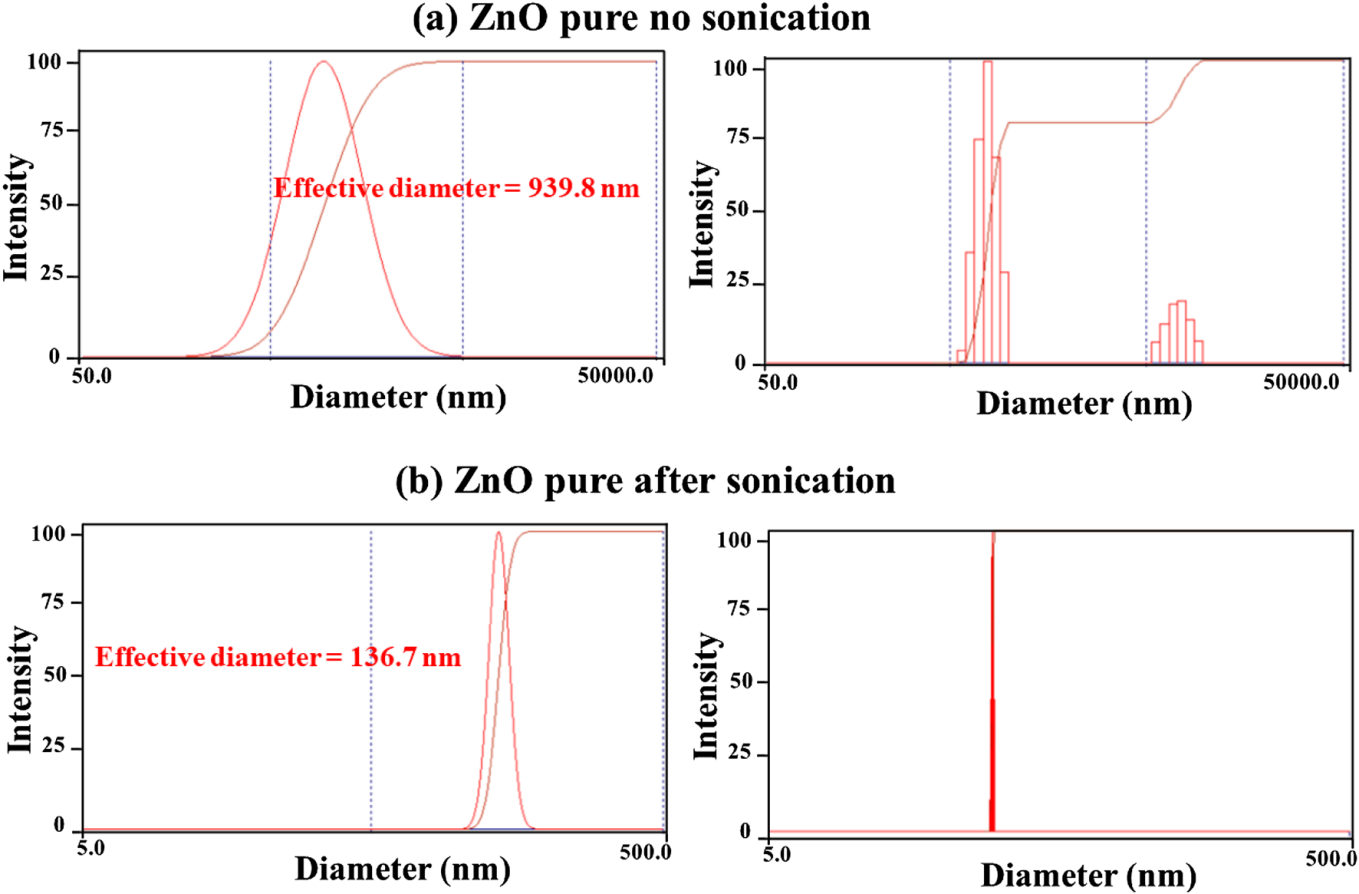
Results of the particle size distribution measurements for the pure ZnO powder without and after sonication.

**Figure 5:**
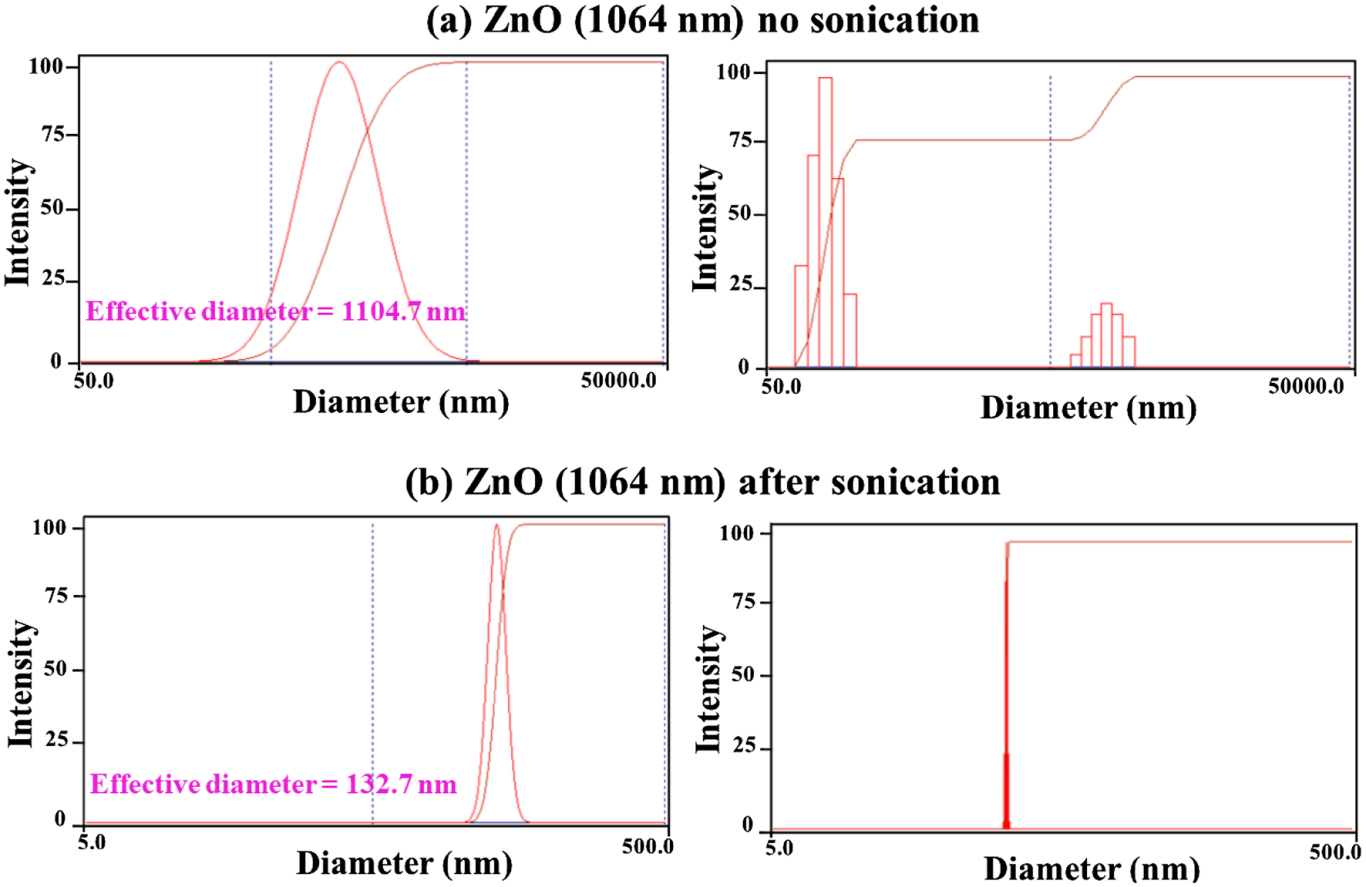
Results of the particle size distribution measurements after being irradiated with laser (1064 nm) for the ZnO powder without and after sonication.

**Figure 6:**
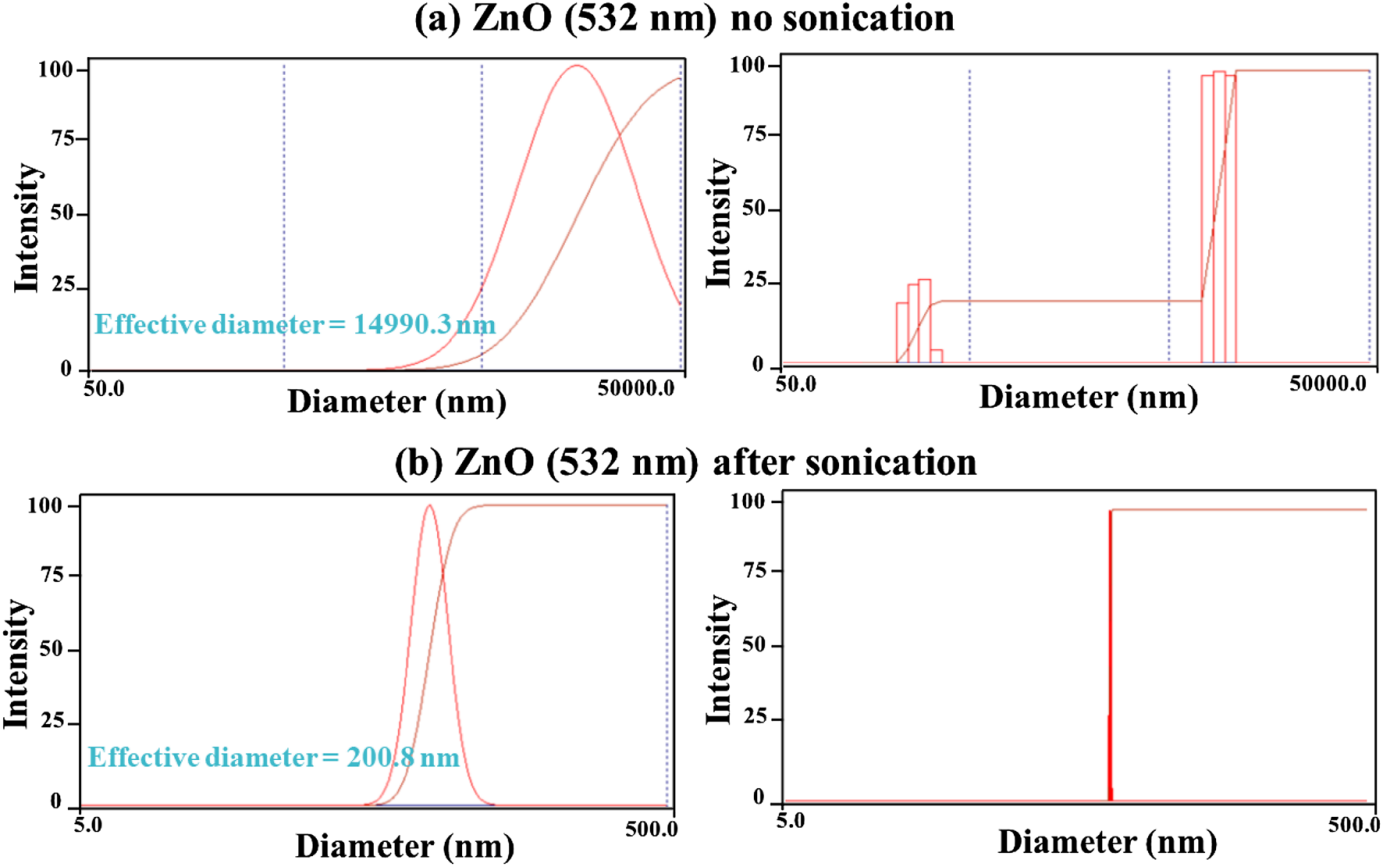
Results of the particle size distribution measurements after being irradiated with laser (532 nm) for the ZnO powder without and after sonication.

Figures 4,5,6) show the particle size distributions of the ZnO nanoparticles before and after being irradiated with laser and with extended sonication. The effective mean diameter was found equal to $136.7 nm$, $132.7 nm$ and $200.8 nm$ for the ZnO nanoparticles before and after irradiation with the $1064 nm$ and $532 nm$ lasers respectively. These results are acceptable due to the fact that the measurement itself is sensitive to the agglomeration property of the nanoparticles. This was confirmed from the results obtained with no sonication performed on the specimens (Figs. 4a,5a,6a), which has been equal to $939.8 nm$, $1104.7 nm$ and $14990.3 nm$ for the ZnO nanoparticles before irradiation and after irradiation with the $1064 nm$ and $532 nm$ lasers respectively. It was found that the sizes obtained for the same nanoparticles are remarkably greater than those obtained after sonication. Furthermore, the distribution exhibited two populations rather than a single population as shown in (Figs. 4b,5b,6b). This is again due to the agglomeration of the nanoparticles.

### Effect of energy irradiation on FT-IR spectra

**Figure 7:**
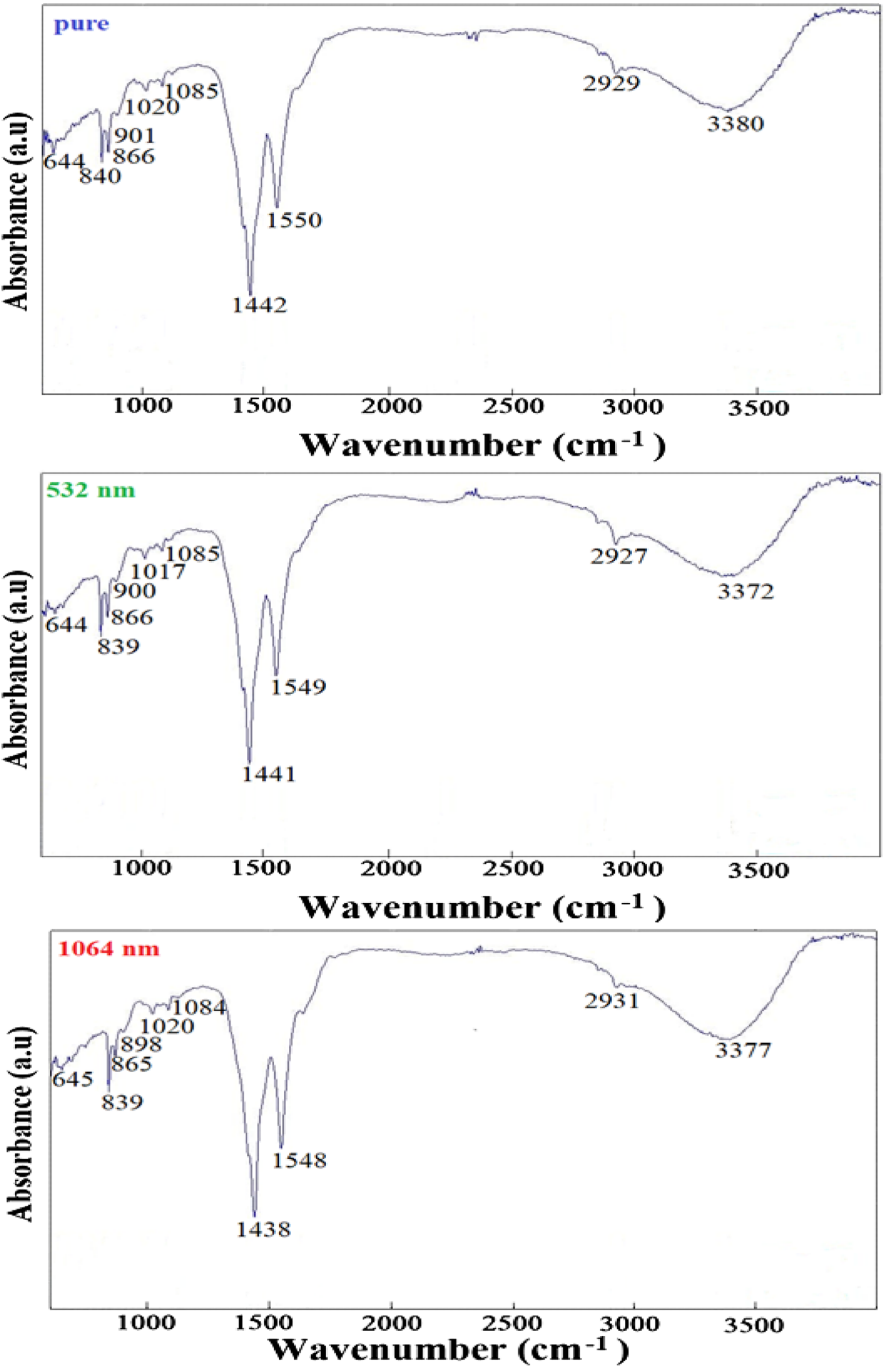
The FTIR Absorbance spectra for the ZnO NPs before and after irradiations.

Results of the FTIR spectroscopy measurements for the ZnO NPs are shown in Fig. 7. The significance of these spectra lies in the possibility of determining the functional group and the vibratory shifts associated with the movement of electrons which appear at specific wavenumbers which is a characteristic feature of each material and compound. An analysis of the FTIR spectra was also performed to identify the active molecules responsible for the inhibition and stabilization of the NPs. The FTIR spectra of the ZnO NPs shows a characteristic peak of the Zn–O bonding at 645.9 cm^−1^. It also shows strong and broad peaks that reflect the complex nature of the O–H bond stretching which is characterized by the high absorption and sharp peak at 3389.48 cm^−1^ (the alcohol group). A medium peak at 1637.78 cm^−1^ was also identified indicating the C=C stretching of the alkene. Intense peaks are also found in the range 898.42—839.64 cm^−1^ indicating the existence of functional groups associated with the C=C bending of alkene, 1548.27 cm^−1^ associated with the N–O bond stretching as nitro compound and a medium peak at 1438.24 cm^−1^ indicating the O–H bending as carboxylic acid.

### Atomic force microscopy images

**Figure 8:**
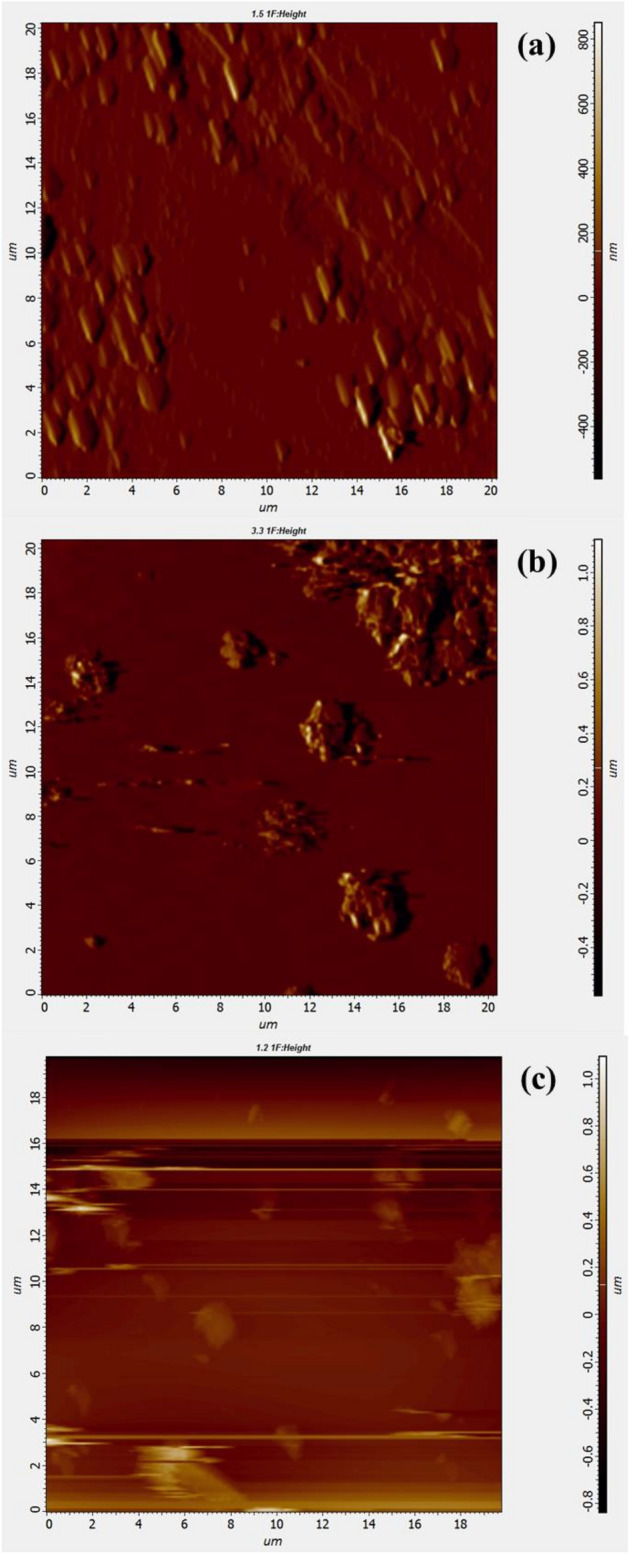
The AFM images of the ZnO NPs before (**a**) and after irradiation with laser having a wavelength 1046 nm (**b**), and 532 nm (**c**).

Figure 8 shows the AFM images of the ZnO NPs in two dimensions before (Fig. 8a), after the irradiation with a laser having wavelengths of $1064 nm$ (Fig. 8b), and after the irradiation with a laser having wavelengths of $532 nm$ (Fig. 8c). The AFM images show the existence of tiny grains of the ZnO NPs with measurable roughness. The grains are also congregating over certain areas of the investigated specimen while other areas show the exposed substrate indicating the tendency of the NPs to agglomerate. The root means square roughness of the non-irradiated and the irradiated nanoparticles with $1064 nm$ and $532 nm$ lasers are $218 nm, 263 nm$ and $316 nm$ respectively. The average roughness is $170 nm, 193 nm$, and $227 nm$ for the non-irradiated and irradiated nanoparticles respectively (i.e. an increase of average roughness with the energy of the laser beam). This might be attributed to the effect of laser on the agglomeration nature of the NPs. This may also be related to the results of the particle size distribution measurements. Nevertheless, one has to refer to the possible sources of errors pertaining to the measurement of roughness using the AFM (i.e. the difficulty in ensuring an even and equivalent distribution of the NPs from the three nanoparticles investigated on the surface of the substrates.

### Effect of energy irradiation on UV–Visible spectra analysis

**Figure 9:**
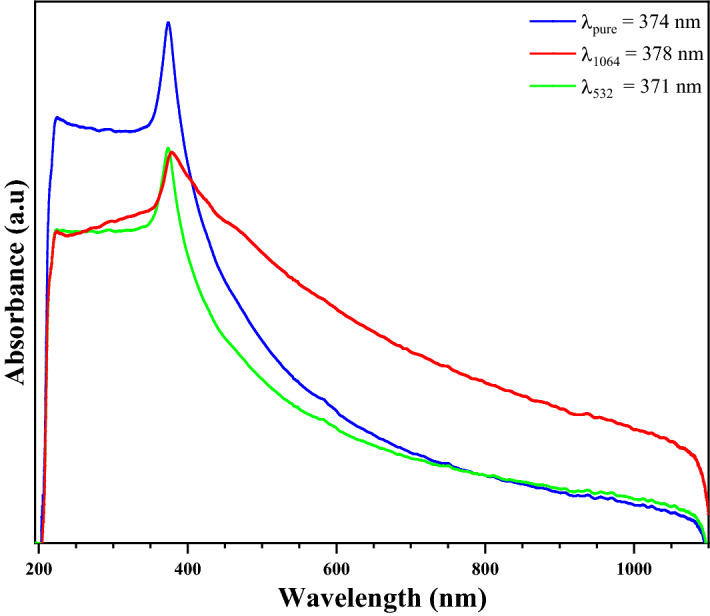
The spectra of the ultraviolet–visible spectroscopy measurements for the ZnO NPs before (blue line) and after irradiations (red and green line).

Figure 9 show ultraviolet–visible (UV–Vis) spectroscopy results of the ZnO. The nanoparticles are known to interact strongly with light at a certain wavelength. UV radiation is therefore an effective tool for the identification and characterization of the structure and absorption of the NPs. As shown in Fig. 9, the spectrum exhibits a maximum near the $374 nm$ wavelength for the non-irradiated NPs. A shift is observed in the spectrum after irradiation with lasers beam having a wavelength $1064 nm$ from $374 nm$ to $378 nm$. But A shift is observed in the spectrum after irradiation with lasers beam having a wavelength $532 nm$ from $374 nm$ to $371 nm$. The reversal of the sense of shift in the maximum absorption is due to the effect of energy of irradiation on the electronic structure of the samples.

### Effect of energy irradiation on the optical parameter

The optical behavior of materials is important to determine its usage in optoelectronic devices, and biomedical application. The optical properties may also be closely related to the material’s atomic structure, electronic band structure and electrical properties. An accurate measurement of the optical constant can be easily performed in inorganic crystals in such crystals, photonic density of states and photonic mode spatial distribution can be tailored with great accuracy.

#### Optical bandgap analysis

The optical absorption measurement near the fundamental absorption edge is a standard method for estimation of the band gap energy. The dependence of optical absorption coefficient with the photon energy helps to study the band structure and the type of transition of electrons. Using the absorption data, the optical absorption coefficient $\left( \alpha \right)$ can be deduced by applying the Beer-Lambert’s law^29^:

12 $$ \alpha = \frac{2.3030 \times A}{e} $$

where $A$ is the maximum absorbance, and $e$ is the sample thickness $\left( {e = 0.003 cm} \right)$.

The inter-band absorption theory shows that, the absorption coefficient near the threshold versus incident energy, is given by the following Pankove’s relation^30^:

13 $$ \alpha h\nu = B\left( {h\nu - E_{g} } \right)^{n} $$

where $B$ is the probability parameter for the transition and $E_{g}$ the optical gap energy. For allowed direct transitions, the coefficient $n$ is equal to $\frac{1}{2}$ and for indirect allowed transitions $n = 2$. Owing to the direct band gap, the crystal under study has an absorption coefficient $\left( \alpha \right)$ obeying the following relation for high photon energies $\left( {h\nu } \right)$:

14 $$ \alpha h\nu = B\left( {h\nu - E_{g} } \right)^{\frac{1}{2}} $$

The $E_{g}$ value corresponding to direct band gap transitions can be calculated via the $\left( {\alpha h\nu } \right)^{2}$ versus $h\nu$, using the formula:

15 $$ \left( {\alpha h\nu } \right)^{2} = B\left( {h\nu - E_{g} } \right) $$

**Figure 10:**
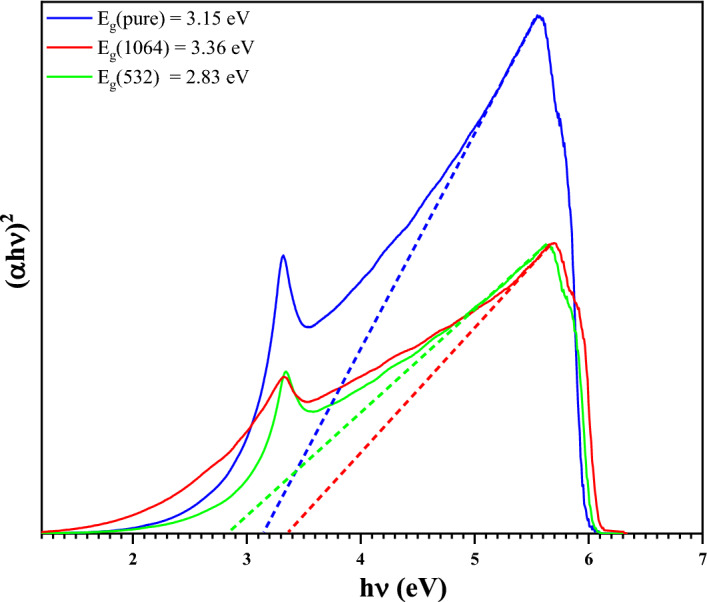
Tauc plot for ZnO NPs before (blue line) and after irradiations (red and green line).

The values of $E_{g}$ were estimated from the intersection of the extrapolated linear part of the $\left( {\alpha h\nu } \right)^{2}$ curves with energy axis. Figure 10 shows the variation of $\left( {\alpha h\nu } \right)^{2}$ versus $h\nu$ before and after irradiation with laser beams for ZnO NPs.

As can be seen Table 5, the bandgap of the pure ZnO NPs is $$3.3155 eV$$, which agrees with the band gap of bulk ZnO. While the ZnO NPs band gap after irradiations by $$1064 nm $$ and $$532 nm$$ has been reduced to $$3.2804 eV$$, and $$3.3423 eV$$ respectively. The laser irradiations provide additional carriers which cause the Fermi level to shift towards the conduction band. Therefore, the width of the bandgap decreases.

**Table 5:** Optical parameter before and after irradiation with laser beams for ZnO nanoparticles.

Samples	${\varvec{\lambda}}_{{{\varvec{max}}}} \left( {{\varvec{nm}}} \right)$	${\varvec{E}}_{{\varvec{g}}} \left( {{\varvec{eV}}} \right)$	${\varvec{n}}$	**A**	${\varvec{\alpha}}\left( {{\varvec{cm}}^{ - 1} } \right)$	${\varvec{\sigma}}_{{{\varvec{opt}}}} \left( { \times 10^{10} \user2{ s}^{ - 1} } \right)$	${\varvec{k}}\left( { \times 10^{ - 4} \user2{ }} \right)$
pure	374	3. 15	2.727	0.865438	664.368	432.519	19.772
Irradiated by 1064 nm	378	3.36	2.678	0.649638	498.705	318.834	15.001
Irradiated by 532 nm	371	2.83	2.812	0.656404	503.899	339.117	14.876

#### Refractive index

The refractive index $\left( n \right)$ is a fundamental parameter of optical materials that plays a very important role in the design of optical devices. Thus, controlling this parameter in polymers makes them practical for a variety of industrial and medical applications. According to the empirical relationship applicable to different varieties of compounds proposed by Reddy et al.^31^, the bandgap value can be used to calculate the refractive index as follows:

16 $$ n = \sqrt {\frac{12.417}{{\sqrt {E_{g} - 0.365} }}} $$

As can be seen Table 5, the small variation of the refractive index value for ZnO, before and after irradiation, indicates that this compound is optically stable and may be a potential candidate for the fabrication of waveguides in which light is transferred in the middle layer by total internal reflection. This only occurs when the refractive index of the middle layer is larger than that of the surrounding layers.

#### Optical conductivity

The optical conductivity $\sigma_{opt}$ for pure ZnO sample was calculated using the absorption coefficient $\alpha$, and the refractive index $n$ data using the following relation^32^:

17 $$ \sigma_{opt} = \frac{\alpha nc}{{4\pi }} $$

where $c$ is the velocity of light in free space. $n$ is the refractive index, and $\alpha$ is absorption coefficient.

The values of the optical conductivity for ZnO NPs, before and after irradiation with laser beams, are in the range $\left( {318 - 433} \right) \times 10^{10} s^{ - 1}$. As can be seen Table 5, the variation of the optical conductivity $\left( {\sigma_{opt} } \right)$ as a function of the energy of the laser irradiation shows that this parameter is proportional to the absorption coefficient $\alpha$, to the bandgap energy $\left( {E_{g} } \right)$ and to the intensity of absorbance $\left( A \right)$. In the spite of the deformation of the electron structure by different energies of irradiation, which has a direct effect on all the optical parameters having as their origin the electronic structure, ZnO presents a good optical stability of their parameters. In addition, that, these high values of the optical conductivity of prepared ZnO NPs pointed out to the superiority of the material for such photovoltaic applications^33^.

#### The extinction coefficient

The extinction coefficient is a measure of the fraction of light lost due to scattering and absorption per unit distance of the penetration medium. It can be estimated from the values of $\alpha$ and $\lambda$ using the relation:

18 $$ k = \frac{\lambda \alpha }{{4\pi }} $$

**Figure 11:**
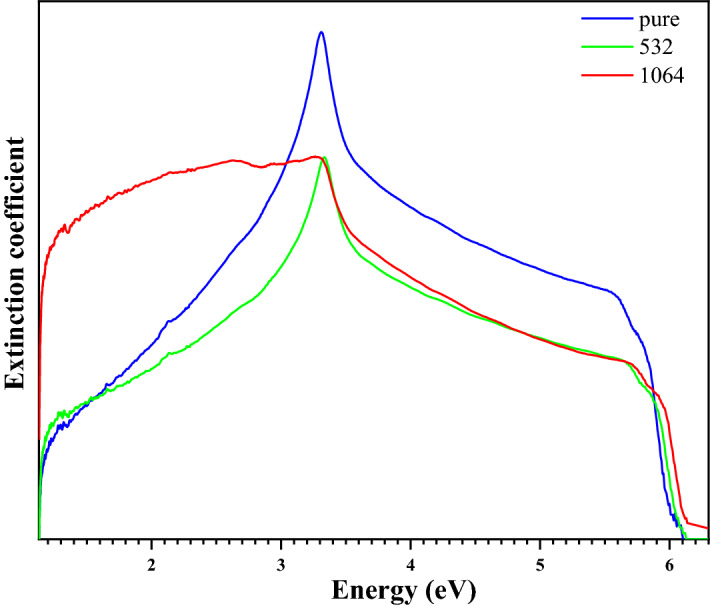
Calculated extinction coefficient plot before (blue line) and after irradiation with laser beams (red and green line) for ZnO NPs.

The extinction coefficient as a function of energy photon is shown in Fig. 11. It can be noted that the extinction coefficient of ZnO decreases by increasing the energy of irradiation (Table 5). The decrease in the extinction coefficient with the increase in wavelength may be correlated with the increase in the transmittance and decrease in the absorption coefficient. The decrease in the extinction coefficient with an increase in wavelength shows that the fraction of light lost due to scattering and absorbance decreases. This property can be used to implement an all-optical switch.

### Effect of energy irradiation on the antibacterial activity

**Figure 12:**
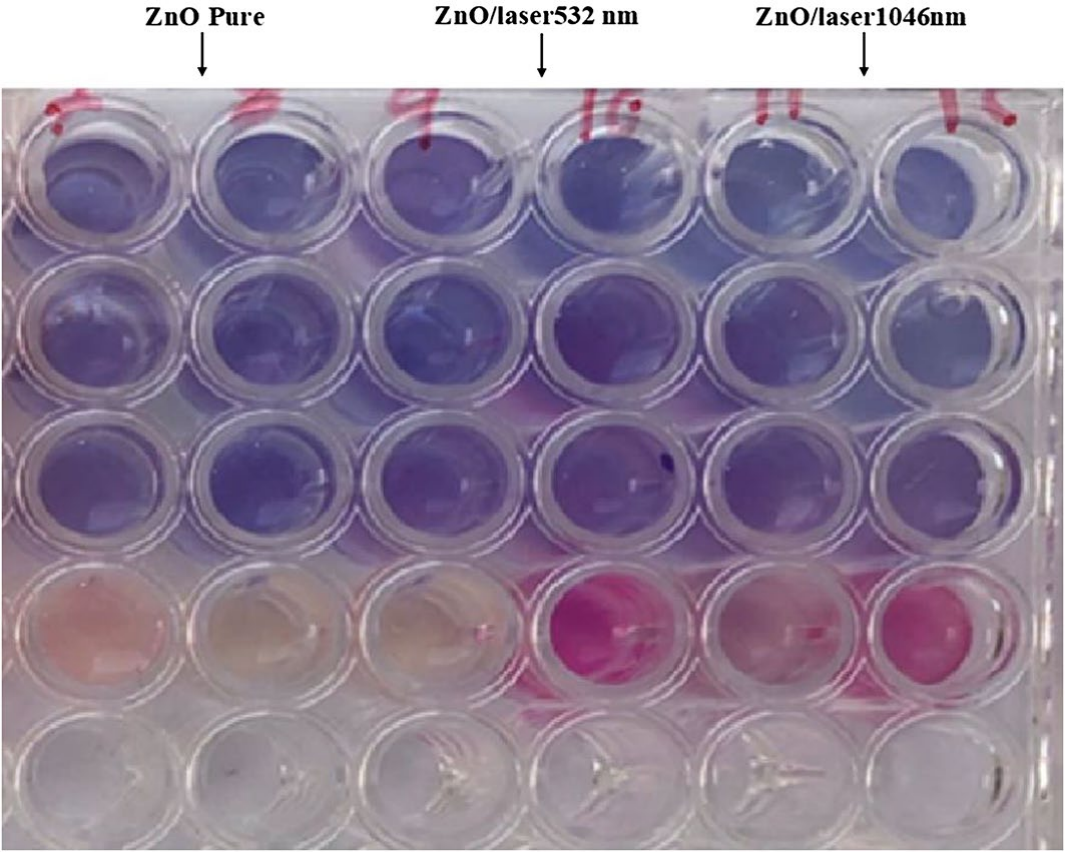
The results showed the effectiveness of ZnO-NPs against bacteria.

The results showed the effectiveness of ZnO-NPs against pseudomonas erogenous (gram negative bacteria) and staphylococcus (gram positive bacteria) at all concentrations used Where when the ZnO-NPs was used with the antibiotic by a synergistic action, this led to an increase in the efficiency of the antibiotic against the target bacteria. As previous studies showed the potential of ZnO-NPs to improve the efficacy of antibiotics against multidrug resistant A. baumannii along with their effect on membrane permeability. To the best of our knowledge, this is the first report on the mechanism of action of ZnO-NPs on the efficiency of ciprofloxacin and ceftazidime antibiotics against resistant A. baumannii. ZnO-NPs alone and in combination with both antibiotics proved to be effective in the control of A. baumannii. In addition, the increased antibacterial activity of ceftazidime and ciprofloxacin in the presence of ZnO-NPs may be due to membrane damage and accumulation of antibacterial agents in the cells^34^. The exact mechanism of antibacterial action of ZnO-NPs with antibiotics has not yet been clearly elucidated. The synergistic activity between conventional antibiotics and NPs has been suggested to be due to inhibition of the export of antibacterial agents by blocking efflux pumps or by enhancing the entrance of antibiotics into the cell by disrupting the bacterial membrane^35^. Several previous studies concluded that ZnO-NPs may induce genotoxicity indirectly by promoting oxidative stress or by directly passing through the cellular membrane and interacting with the DNA to damage all four bases or producing thymine–tyrosine cross-linking^36^ (see Fig. 12).

**Figure 13:**
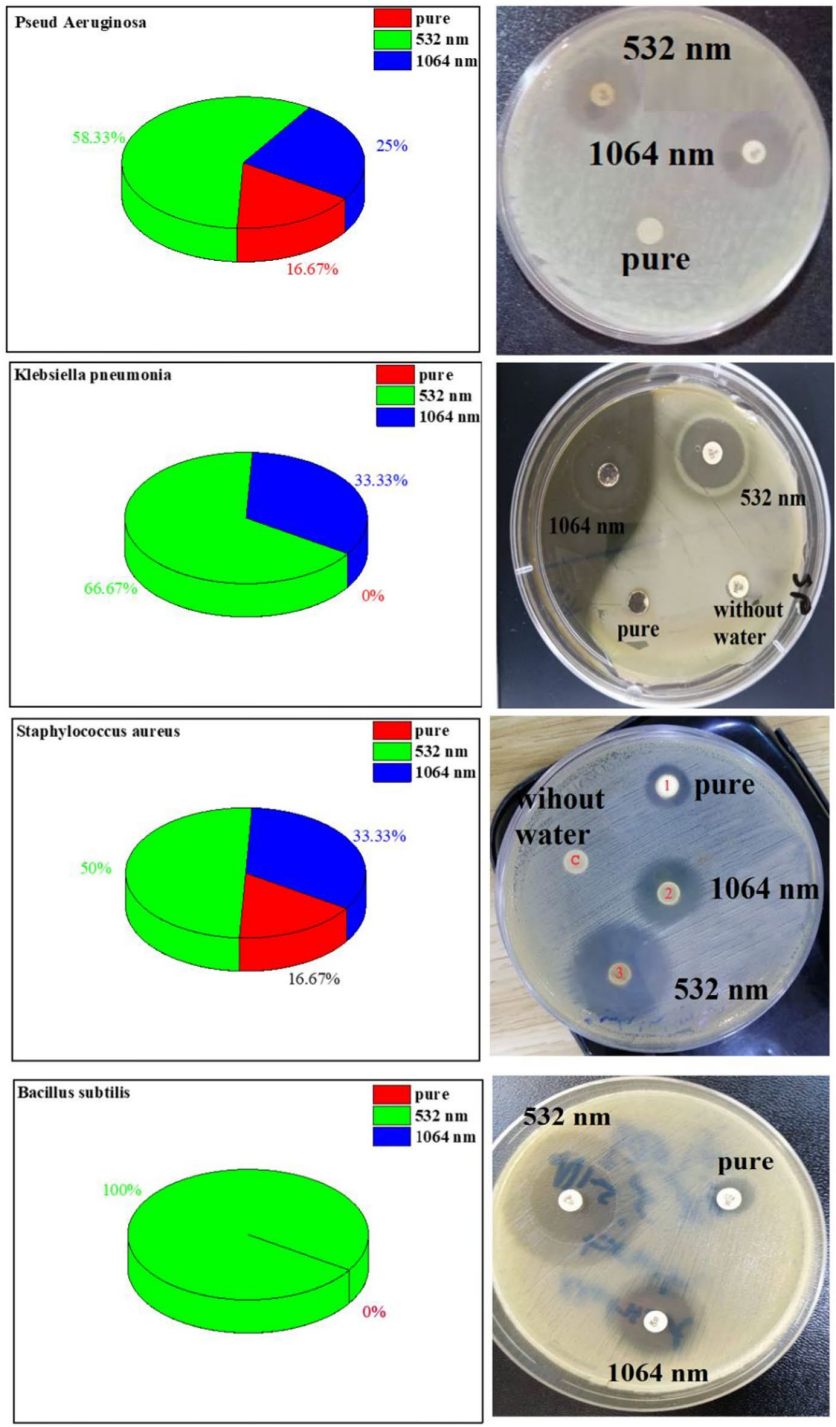
Inhibition ZnO-NPs pure, before and after irradiation by laser with bacteria.

The area of inhibition when using ZnO-NPs on a mullar Hinton Agar medium against pseudomonas erogenous (gram negative bacteria) and staphylococcus (gram positive bacteria), which showed the highest inhibition when using ZnO-NPs treated with wavelength $532 nm$ $\left( {12 mm} \right)$, followed by a wavelength 1064 nm where the area of inhibition was (8 mm) and the results showed the least inhibition when using pure ZnO-NPs without any treatment was $\left( {4 mm} \right)$. See Fig.13 and Table 6 our results are agreement with this source^36^.

**Table 6:** Mean inhibition Zones of the synthesized ZnO NPs before and after irradiation by laser beam (532 nm and 1064 nm).

Bacterial strains	Diameter zone inhibition (mm)			
	Without water	In water		
	c	Pure	532 nm	1064 nm
Pseud Aeruginosa	0	2	7	3
Klebsiella pneumonia	0	0	8	4
Staphylococcus aureus	0	4	12	8
Bacillus subtilis	0	0	9	0

Their effects were more pronounced on Gram-positive bacteria than Gram-negative bacteria. Consistent with the findings, it can be summarized that ZnO-NPs are considered as effective antibacterial agents against both Gram-positive and Gram-negative bacteria^37^.

To the best of our knowledge, the structure of the outer layer of Gram-positive bacteria is composed of peptidoglycan; meanwhile, Gram-negative bacteria are composed of phospholipids, and both undergo different interactions when exposed to ZnO-NPs. Several studies suggested some possible antibacterial mechanisms of ZnO-NPs; they easily penetrated bacterial cells and released zinc ions (Zn2 +) that inhibit active transport, bacterial enzymes required for metabolic activity and finally induced cell death because of the huge reactive surface of NPs. The other proposed that the formation of reactive oxygen species (ROS) generated from the surface of NPs leads to oxidative stress and subsequent cell damage^38^.

In another way their potential in vitro antibacterial effects against most common bacterial pathogens infection Nile tilapia directs our vision for possible in vivo application to control the invading and emergent pathogens^39^.

## Conclusions

ZnO NPs were successfully synthesized by sol–gel method. The as-synthesized NPs were irradiated with lasers having wavelengths of $1064 nm$ and $532 nm$ to improve their structural and optical properties. The structural and elemental analysis of the NPs emphasized the formation of high aggregates of high-purity ZnO NPs in the range $42-46 nm$ having hexagonal crystalline structure. The ZnO nanoparticles shows no change in the preferred orientation after irradiation with laser, and no additional peaks are observed after irradiation with laser indicating the high crystalline quality of the material. A shift is observed in the UV–Visible spectra after irradiation due to the effect of irradiation on the electronic structure of the samples. In the spite of the deformation of the electron structure by different energies of irradiation, which has a direct effect on all the optical parameters having as their origin the electronic structure, ZnO presents a good optical stability of their parameters allows the synthesized compound to be a good candidate for optoelectronic, and photonics applications.

The irradiation of ZnO nanoparticles by laser beams of wavelength 1064 nm and 532 nm decreases the size of the crystallites which increases their antibacterial activity.

The efficacy of the antibiotic's ciprofloxacin and ceftazidime on gram-negative bacteria and gram-positive bacteria was found to improve with the addition of the ZnO NPs synthesized after their irradiations with 532 nm laser beams and with less effect for 1064 nm.

## Data Availability

All data generated or analyzed during this study are included in this published article.

## References

[1] Beaucage, G., et al. Nanostructured powders and their industrial application. *Mater. Res. Soc. Symp. Proc.* **520**, (1998).

[2] Global Information Inc. **2005**, Nanomaterials (2008).

[3] Freedonia Group, CT, USA, RNCOS E-Services Pvt. Ltd. (2008).

[4] Nanotechnology Market Forecast (2011).

[5] Research Report, CanBiotech Inc., Canada Electronics ca. Publications (2008).

[6] Nanomaterials and Markets, Technology Transfer Centre, Research Report #INT6571, p.245, (2015).

[7] Cho KH (2005). The study of antimicrobial activity and preservative effects of Nano silver ingredient. Electrochim. Acta.

[8] Djoko KY (2015). The role of copper and zinc toxicity in innate immune defense against bacterial pathogens. J. Biol. Chem..

[9] Sirelkhatim A (2015). Review on zinc oxide nanoparticles: Antibacterial activity and toxicity mechanism. Nano-Micro Letters..

[10] Gemma CC (2019). Antibacterial nanoparticles. Comprehensive Nanosci. Nanotechnol..

[11] Ali A (2018). Elemental zinc to zinc nanoparticles: is ZnO NPs crucial for life? Synthesis, toxicological, and environmental concerns. Nanotechnol. Rev..

[12] Manzoor U (2015). Effect of synthesis temperature, nucleation time, and post synthesis heat treatment of ZnO nanoparticles and its sensing properties. J. Nanomater..

[13] Gunjan B (2016). Synthesis, characterization, and study of in vitro cytotoxicity of ZnO-Fe_3_O_4_ magnetic composite nanoparticles in human breast cancer cell line (MDA-MB-231) and mouse fibroblast (NIH 3T3). Nanoscale Res. Lett..

[14] Bettini S (2016). Enhancement of open circuit voltage of a ZnO-based dye-sensitized solar cell by means of piezotronics effect. Chem. Asian J..

[15] Spanhel L, Anderson MA (1991). Semiconductor clusters in the sol-gel process: Quantized aggregation, gelation, and crystal growth in concentrated zinc oxide colloids. J. Am. Chem. Soc..

[16] Haque MdJ (2020). Synthesis of ZnO nanoparticles by two different methods & comparison of their structural, antibacterial, photocatalytic and optical properties. Nano Express.

[17] Zhang Y (2013). Biomedical applications of zinc oxide nanomaterials. Curr. Mol. Med..

[18] Vanheusden K (1996). Mechanisms behind green photoluminescence in ZnO phosphor powders. J. Appl. Phys..

[19] Desarkar HS (2013). One-step synthesis of Zn/ZnO hollow nanoparticles by the laser ablation in liquid technique. Laser Phys. Lett..

[20] Yamashita T (2006). The relationships between microstructure and crystal structure in zincite solid solutions. J. Mater. Sci..

[21] Li J-L, Gu M (2010). Gold-nanoparticle-enhanced cancer photothermal therapy. IEEE J. Sel. Top. Quantum Electron.

[22] Solati E (2014). Effect of laser pulse energy and wavelength on the structure, morphology and optical properties of ZnO nanoparticles. Opt. Laser Technol..

[23] Suryanarayana C, Norton MG (1998). X-ray diffraction a practical approach.

[24] Shkir M (2017). Facile microwave-assisted synthesis of tungsten-doped hydroxyapatite nanorods: A systematic structural, morphological, dielectric, radiation and microbial activity studies. Ceram. Int..

[25] Cullity BD (1978). Elements of X-ray Diffraction.

[26] Seetawan U (2011). Effect of calcinations temperature on crystallography and nanoparticles in ZnO disk. Mater. Sci. Appl..

[27] Pandiyarajan T, Karthikeyan B (2012). Cr doping induced structural, phonon and excitonic properties of ZnO nanoparticles. J. Nanopart. Res..

[28] Pal U (2006). Synthesis and optical properties of ZnO nanostructures with different morphologies. Opt. Mater..

[29] Jadhav SR, Khairnar UP (2012). Study of optical properties of Co-evaporated PbSe thin films. Archive Appl. Sci. Res..

[30] Almudhaffer MF (2012). Linear optical properties and energy loss function of Novolac: Epoxy blend film. Archive Appl. Sci. Res..

[31] Reddy RR, Ahammed YN (1995). A study on the Moss relation. Infrared Phys. Technol..

[32] Abdallah OGh (2015). Reducing the optical band gap of polyvinyl alcohol (PVA) based nanocomposite. J. Mater. Sci. Mater. Electr..

[33] Gratzel M (2014). The light and shade of perovskite solar cells. Nat. Mater..

[34] Ghasemi F, Jalal R (2016). Antimicrobial action of zinc oxide nanoparticles in combination with ciprofloxacin and ceftazidime against multidrug-resistant Acinetobacter baumannii. J. Global Antimicrobial Resistance..

[35] Banoee M (2010). ZnO nanoparticles enhanced antibacterial activity of ciprofloxacin against Staphylococcus aureus and Escherichia coli. J. Biomed. Mater. Res. B Appl. Biomater..

[36] Sharma V (2009). DNA damaging potential of zinc oxide nanoparticles in human epidermal cells. Toxicol. Lett..

[37] Meruvu H (2011). Synthesis and characterization of zinc oxide nanoparticles and its antimicrobial activity against Bacillus subtilis and *Escherichia coli*. Rasayan J. Chem..

[38] Agarwal H (2018). Mechanistic study on antibacterial action of zinc oxide nanoparticles synthesized using green route. Chem. Biol. Interact..

[39] El-Saadony MT (2021). Impact of mycogenic zinc nanoparticles on performance, behavior, immune response, and microbial load in Oreochromis niloticus. Saudi J. Biol. Sci..

